# Surface Electromyographic Examination of Poststroke Neuromuscular Changes in Proximal and Distal Muscles Using Clustering Index Analysis

**DOI:** 10.3389/fneur.2017.00731

**Published:** 2018-01-15

**Authors:** Weidi Tang, Xu Zhang, Xiao Tang, Shuai Cao, Xiaoping Gao, Xiang Chen

**Affiliations:** ^1^Department of Electronic Science and Technology, University of Science and Technology of China, Hefei, China; ^2^Department of Rehabilitation Medicine, First Affiliated Hospital of Anhui Medical University, Hefei, China

**Keywords:** muscle weakness, clustering index, surface electromyography, neuromuscular changes, stroke rehabilitation

## Abstract

Whether stroke-induced paretic muscle changes vary across different distal and proximal muscles remains unclear. The objective of this study was to compare paretic muscle changes between a relatively proximal muscle (the biceps brachii muscle) and two distal muscles (the first dorsal interosseous muscle and the abductor pollicis brevis muscle) following hemisphere stroke using clustering index (CI) analysis of surface electromyograms (EMGs). For each muscle, surface EMG signals were recorded from the paretic and contralateral sides of 12 stroke subjects versus the dominant side of eight control subjects during isometric muscle contractions to measure the consequence of graded levels of contraction (from a mild level to the maximal voluntary contraction). Across all examined muscles, it was found that partial paretic muscles had abnormally higher or lower CI values than those of the healthy control muscles, which exhibited a significantly larger variance in the CI *via* a series of homogeneity of variance tests (*p* < 0.05). This finding indicated that both neurogenic and myopathic changes were likely to take place in paretic muscles. When examining two distal muscles of individual stroke subjects, relatively consistent CI abnormalities (toward neuropathy or myopathy) were observed. By contrast, consistency in CI abnormalities were not found when comparing proximal and distal muscles, indicating differences in motor unit alternation between the proximal and distal muscles on the paretic sides of stroke survivors. Furthermore, CI abnormalities were also observed for all three muscles on the contralateral side. Our findings help elucidate the pathological mechanisms underlying stroke sequels, which might prove useful in developing improved stroke rehabilitation protocols.

## Introduction

Muscle weakness is the most common clinical symptom of many neuromuscular diseases (such as stroke and spinal cord injury) and it greatly impacts the day-to-day quality of life for patients and their caregivers (1, 2). Stroke studies have reported that weakness related to voluntary muscle contraction is a primary cause of impairments, including spastic hypertonia and abnormal movement coordination (3). This contributes to impaired motor control (4). Therefore, it is of great importance to understand the specific pathological mechanisms underlying muscle weakness after a stroke, which is a prerequisite for designing effective stroke rehabilitation protocols.

The interruption of the corticospinal tract and muscle atrophy are commonly accepted as two potential contributors to the muscle weakness of stroke survivors (5, 6). How a cerebral lesion affects motor unit (MU) survival and function, however, still remains ambiguous. Since the MU offers a structure–function framework for understanding the neuromuscular system, investigations into MU alternations provide valuable insights into the neuropathology of stroke-induced muscle weakness. Many muscle biopsy studies have reported various contradictory findings. For example, little difference was found between the paretic side and contralateral side of stroke survivors even within populations of stroke subjects and control subjects (7, 8). However, some other muscle biopsy studies showed atrophy of type II fibers, small angular fibers, grouped atrophy, and fiber-type grouping (9, 10), which all indicated degeneration of MUs. The same results were observed in electrophysiological studies. Some studies have found spontaneous fibrillation potentials and positive sharp waves in paretic muscles (6, 9, 11). Furthermore, reduced compound muscle action potentials and MU number estimates were also reported (12–14). Nevertheless, other electrophysiological studies did not report consistent findings (15–17). These studies were associated with either invasive procedures or laborious electrical stimulations. In contrast, surface electromyogram (EMG) is an alternative approach for examining MU alternations in a noninvasive manner. Surface EMG studies in stroke patients have been previously performed through an EMG-force relation (18–20), peak amplitude distribution (21), and power spectral analysis (22). These studies have reported mixed observations, suggesting that there are a variety of complex neural and muscular changes collectively contributing to muscle weakness following a stroke (23–25).

However, different choices with respect to the muscles examined might be another explanation for the previously mixed observations. Muscles in various body parts often show different changes poststroke. For example, in an experiment involving simultaneous flexion of proximal and distal paretic muscles of stroke survivors, which included the deltoids, biceps, and wrist/finger flexors, the wrist/finger flexors had the lowest coefficient of variation (26). Furthermore, the finger flexor was also reported to have a higher motor unit action potential (MUAP) median frequency and larger range of MUAP RMS amplitude than biceps brachii (BB) muscles following stroke (27). One possible hypothesis to explain this is that the pathological MU alternation may take place in different muscles to different degrees and this might vary from proximal to distal positions. Therefore, it is necessary to explore whether there are differences in the MU alternation between proximal and distal muscles on the paretic sides of stroke survivors.

This study presented a novel study of the proximal and distal muscles of stroke subjects using the clustering index (CI) method. The CI method, originally proposed by Uesugi et al. (28), is applied on surface EMG signals to quantitatively assess the clustering degrees for the signals. The clustering or density degree of surface EMG signal, as characterized by the value of CI, can be a useful indicator of neuromuscular changes. It was found that highly clustered EMG interference patterns can be a sign of neurogenic changes, while flat and dense EMG interference patterns might indicate myopathic changes (28–30). Thus, the CI method has strong diagnostic power in differentiating neurogenic and myopathic changes (28). Taking advantage of such a powerful tool, we aimed to discriminate neurogenic and/or myopathic changes taking place in the paretic muscles of stroke survivors and we compared these changes among the proximal and distal muscles examined. Similarities or differences between the proximal and distal muscles after a stroke, if discernable by a CI analysis, might help to understand the specific pathological mechanisms underlying muscle weakness. This would contribute to the development of a more accurate rehabilitation protocol targeting different muscles.

## Materials and Methods

### Subjects

Twelve stroke subjects (S1–S12, age: 63 ± 12 years, mean ± SD, range: 46–82 years) and eight age-matched healthy control subjects (C1–C8, age: 58 ± 10 years, range:48–75 years) were recruited for this study, which was approved by the Medical Ethics Review Committee at the First Affiliated Hospital of Anhui Medical University (FAHAMU, Hefei, Anhui Province, China). Study inclusion criteria included: (1) experience of first stroke with an initial onset >14 days; (2) medically stable with clearance to participate; (3) experience of hemiparesis with mild to severe muscle weakness on the paretic side; (4) ability to fully or partially perform voluntary contraction of the three examined muscles on the paretic side, including the BB muscle, the first dorsal interosseous (FDI) muscle, and the abductor pollicis brevis (APB) muscle; (5) no history of severe muscle spasticity for the three examined muscles based upon a modified Ashworth scale not exceeding 1 for any muscle; and (6) no history of concurrent neurological disorders or other symptoms (such as neuropathy or radiculopathy). All stroke subjects were recruited from the inpatient department of rehabilitation medicine in the FAHAMU. Clinical assessments performed prior to each patient’s participation included both an assessment of motor recovery after a stroke based on the Brunnstrom stage and the upper-extremity component of the Fugl-Meyer test. Detailed information pertaining to the stroke subjects is presented in Table 1. Written consent was obtained from all subjects before the initiation of experiments.

**Table 1:** Demographic information for each stroke subject.

ID #	Age in a range (years)	Duration (days)	Paretic side	B stage	F-M	MAS
S1	75–80	27	L	3	33	0
S2	71–75	89	L	2	15	0
S3	51–55	153	L	5	45	0
S4	81–85	34	L	5	55	0
S5	45–50	30	L	4	43	0
S6	55–60	71	L	4	21	0
S7	71–75	31	L	4	39	0
S8	55–60	47	R	3	38	0
S9	51–55	51	R	3	42	0
S10	61–65	28	L	2	29	0
S11	81–85	24	R	5	37	0
S12	45–50	45	L	4	34	0

*B stage, Brunnstrom stage; F-M, upper-extremity Fugl-Meyer assessment; MAS, Modified Ashworth Scale*.

### Experiments

**Figure 1:**
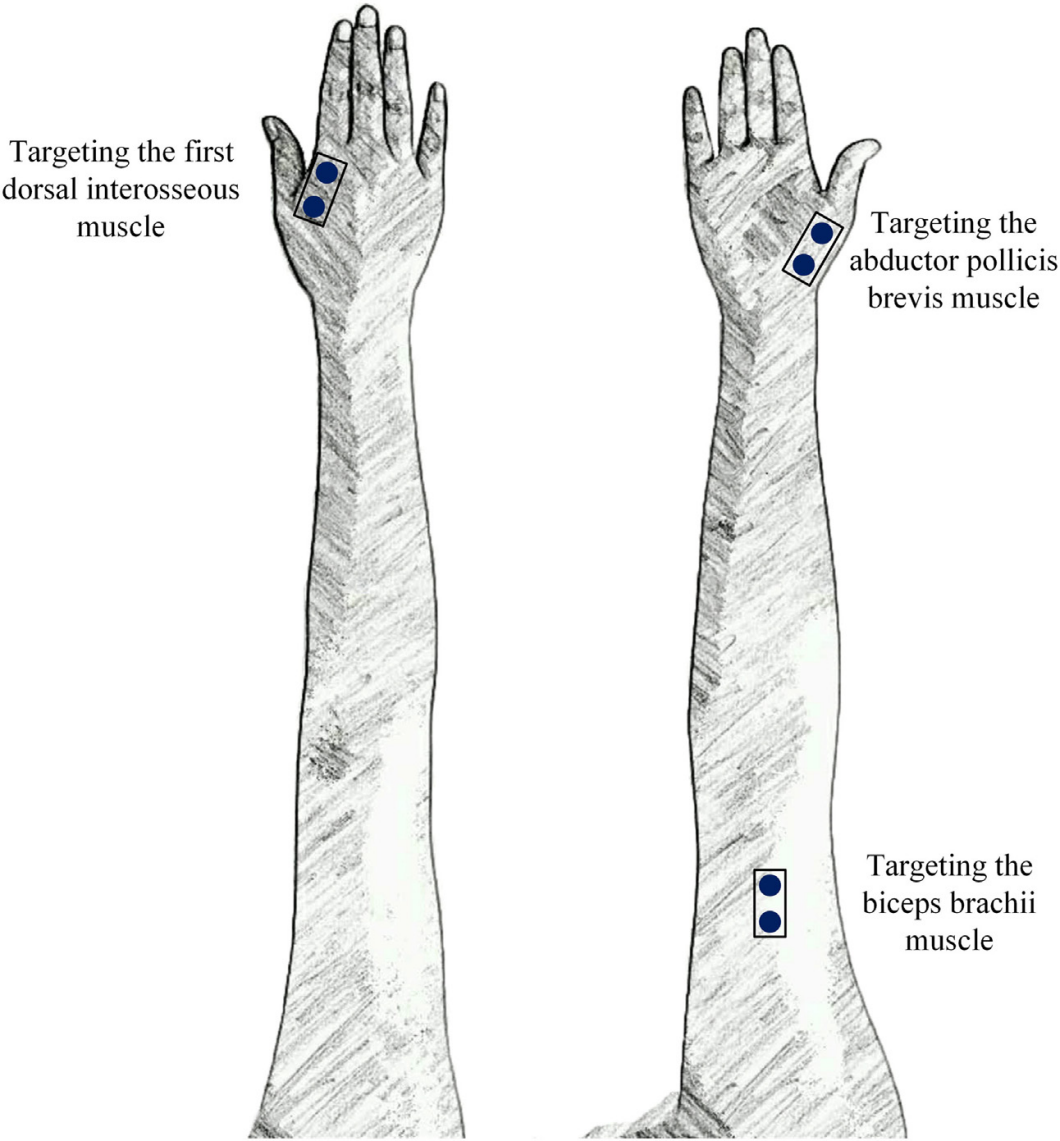
The placement of three surface electromyogram (EMG) sensors that targeted three muscles in each arm.

Ag/AgCl disc surface electrodes (Junkang, Shanghai, China) with a recording diameter of 10 mm were used for recording surface EMG signals. The surface EMG data were collected from the biceps, FDI muscle, and APB muscle on both sides of all stroke subjects and on the dominant side of all control subjects. After the skin was prepared with medical alcohol, a pair of electrodes was firmly attached to each targeted muscle and oriented in the direction of the muscle fibers with an inter-electrode distance (center-to-center distance) of about 20 mm to produce a single-differential channel of surface EMG signals. A round electrode was placed on an arm fossa cubitalison on the same side as a reference. Figure 1 illustrates the electrode placement on the right arm as an example.

During the experiment, subjects were seated in a comfortable chair with their tested arm bent approximately 90° and placed on a height-adjustable table. The stroke patients were also allowed to lie in an examination bed with their tested arm held still and placed against the inside of the body. The recordings were performed on each side of the subject in random order.

For each subject, the experiment was carried out in multiple trials. In each trial, the subjects were asked to perform a specific task according to the main function of the muscle being examined. We designed specific tasks to examine the APB, FDI, and BB muscles, including adducting the thumb, abducting the index finger, and elbow flexion, respectively. In a single trial, each task was performed as isometric muscle contractions with different contraction levels ranging from a mild to submaximal to, ultimately, maximal voluntary contractions (MVC). The contraction strengths roughly corresponded to 10, 30, 50, 70, and 90% of the full MVC. The contraction strengths were subjectively determined by each subject. In this regard, the MVC presented here was not accurately measured, but instead roughly estimated as the maximal level that each subject was encouraged to reach. At the same time, a resistance force was provided by the experimenter to help generate an isometric contraction. The corresponding resistance force was almost equal to the force provided by each subject. In order to examine the APB muscle, a resistance force on the tested thumb was generated. As for the FDI muscle, a resistance force was applied on the index finger. When the BB muscle was examined, the subjects were instructed to perform elbow flexion with their elbow flexed at 90°, while a resistance force was applied on the inner side of the forearm. We encouraged the subjects to remain as stable as possible for at least 3 s during each type of contraction in order to ensure that the interference EMG patterns recorded for each trial exhibited a sequence of graded contraction levels. For each task, multiple (almost 3) trials were performed in order to obtain a sufficient amount of data. Sufficient rest was allowed between consecutive trials to avoid mental and muscular fatigue.

Surface EMG data were recorded using a custom-made data acquisition system supporting up to 128 EMG channels. Each recorded EMG channel was amplified by a two-stage amplifier with a total gain of 60 dB, which was band-pass filtered at 20–500 Hz and subsequently converted into digitalized data with a 16-bit A/D converter. The sampling rate for each channel was set to 1,000 Hz. All recorded data were transferred to a laptop computer *via* a USB cable for off-line analysis in Matlab (The Mathworks, MA, USA) using customized programs.

### Data Analysis

Each surface EMG channel was preprocessed using a zero-lag fourth-order Butterworth band-pass filter at 20–500 Hz to eliminate low-frequency motion artifacts and high-frequency interference. If necessary, a set of second-order notch filters at the 50-Hz power line interference and its harmonics were also applied.

Since we collected data from three different muscles, the following data analyses were performed on individual muscles. Specifically, for each muscle, data segmentation was done after preprocessing. According to the experimental protocol, the recorded surface EMG data in each trial generated graded interference patterns. Thus, a series of non-overlapping epochs with a 1-s duration were segmented from the recorded data, using a straightforward scheme based on the signal amplitude thresholding (31). These epochs were selected from stable isometric muscle contractions. Those epochs with varying contraction strengths were discarded. We obtained approximately 10 epochs from each trial, including epochs at different force strengths. Finally, for each muscle, we obtained approximately 30 epochs from different force strengths. The following CI analysis was performed on these epochs from each muscle.

In order to calculate CI values, the signal for each epoch was divided into a series of non-overlapping consecutive windows of the same length. We set the window length as 15 ms, which was considered to include approximately one individual MUAP in this study (28–30). We assumed that there were *k* windows derived in an epoch, and the area of each window was *A_i_*. The differential sequences for the area values between every consecutive window (DA*_i_*), every second window (DB*_i_*), and every third window (DC*_i_*) were defined as follows:

(1) $${\text{DA}}_{i}=A_{i+1}-A_{i},\text{ for }i=1,2,\ldots ,k-1,$$

(2) $${\text{DB}}_{i}=A_{i+2}-A_{i},\text{ for }i=1,2,\ldots ,k-2,$$

(3) $${\text{DC}}_{i}=A_{i+3}-A_{i},\text{ for }i=1,2,\ldots ,k-3.$$

Consequently, we can calculate CI according to the following equation:

(4) $$\text{CI}=\left(\sum_{i=1}^{k-1}{\text{DA}}_{i}^{2}+\sum_{i=1}^{k-2}{\text{DB}}_{i}^{2}+\sum_{i=1}^{k-3}{\text{DC}}_{i}^{2}\right)/6\times \sum_{i=1}^{k}A_{i}^{2}.$$

The CI values ranged from 0 to 1, while the higher values were derived from signals with higher area clustering degrees, which appeared isolated in large action potential spikes (30).

**Figure 2:**
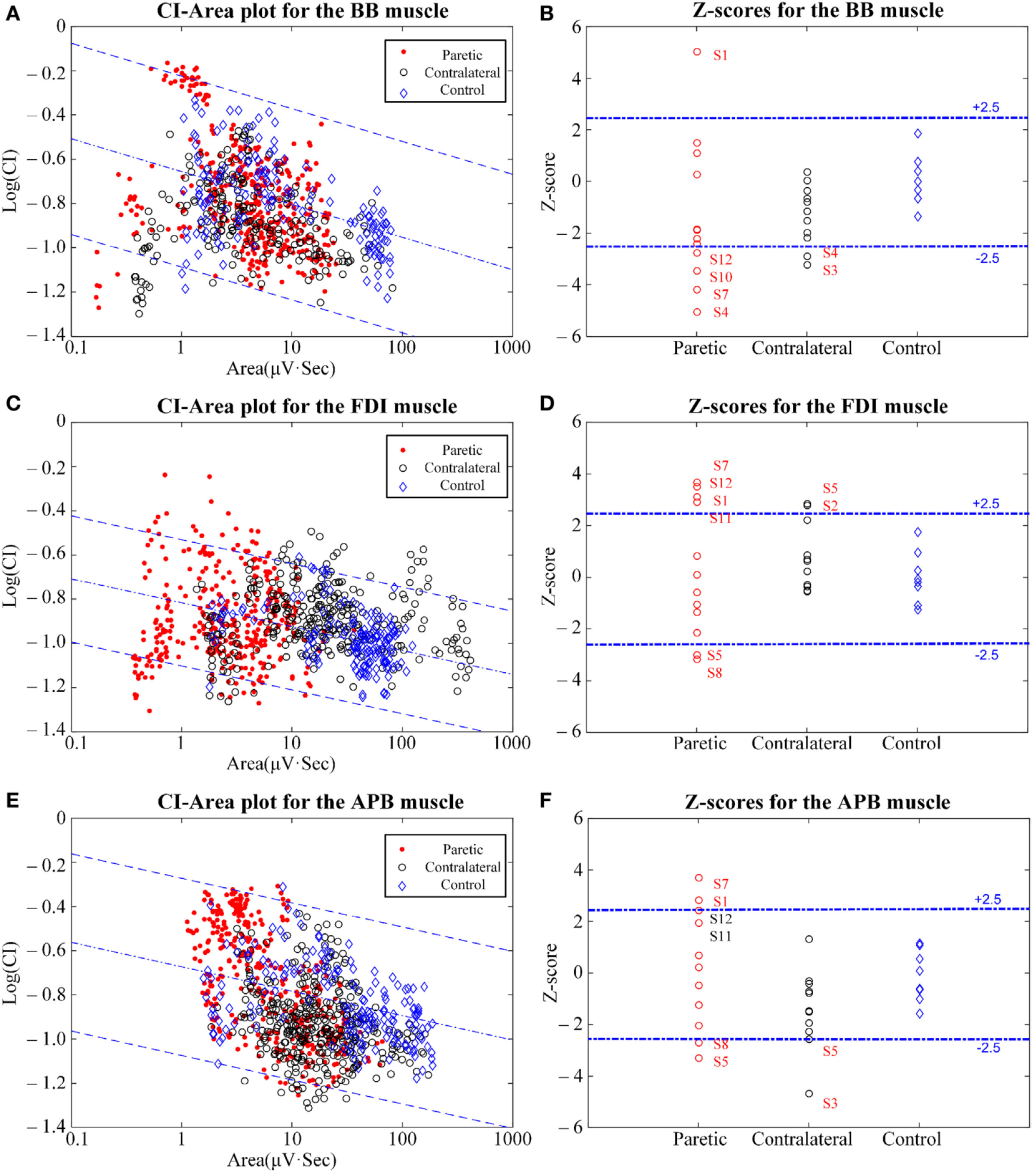
The clustering index (CI)-area plot [**(A,C,E)** left panel] and the resultant *Z*-scores [**(B,D,F)** right panel] using CI analysis of the biceps brachii (BB) muscle **(A,B)**, the first dorsal interosseous (FDI) muscle **(C,D)**, and the abductor pollicis brevis (APB) muscle **(E,F)**. In the CI-area plots, the red dots represent the paretic muscles, the black circles represent the contralateral muscles, and the blue rhombuses represent the control muscles. In the subplots for the *Z*-scores, the muscles with *Z*-scores outside the normal range and some approximating to the normal range are marked with their subject IDs.

Since the CI value is affected by muscle contraction strength, its effect needed to be taken into consideration prior to the establishment of diagnostic criteria. A linear relationship was reported between the CI value and the signal area (representing the muscle contraction strength calculated by $\text{Area}=\sum_{i=1}^{k}A_{i}$) of the epochs from all healthy control subjects using a double-logarithmic scale, and it was recommended by the proposer of the CI method. We found this was also the case for our data and the processed the data in the same way. Therefore, each analysis epoch was expressed as a point in the CI-area plot with the value of log (Area) and log (CI). For each muscle examined, the points derived from all the analysis epochs were scattered to form a data cloud in a CI-area plot. More details can be found in Figure 2 and the Section “Results.”

Quantification of the normal data reference was the prerequisite for identifying muscle abnormalities using the CI method. To define the normal range in the CI-area plot, we performed a linear regression analysis on epochs (1 ≤ Area ≤ 100 μV·s) collected from the corresponding control muscles for both log (CI) and log (Area). Subsequently, for each epoch, the deviation of CI on the logarithmic scale from the linear regression line was calculated. These deviation values were averaged over all the epochs from each examined muscle, and a mean residual (*R*_m_) was obtained. The *R*_m_ was then used to assess the presence of abnormalities for each muscle.

Afterward, the mean μ*_R_* and SD σ*_R_* of the *R*_m_ values for all corresponding muscles of all controls were calculated. On this basis, a *Z*-score for the *R*_m_, which was calculated by (*R*_m_ − μ*_R_*)/ σ*_R_*, was defined for a tested muscle in a given subject. According to the original literature regarding the CI method (28, 30), *Z*-scores were used as the final representative indicator for the diagnostic assessment. We defined a muscle with a *Z*-score outside ±2.5 as abnormal. Assuming that the normal data obey a Gaussian/normal distribution, probability for a *Z*-score outside ±2.5 (with a deviation of 2.5 times SD from the mean) is less than 1.25%. With such a low probability, a *Z*-score outside this range was defined to be abnormal. Further, muscle with a *Z*-score higher than +2.5 was diagnosed as neurogenic, while a *Z*-score lower than −2.5 indicated a myopathic change.

### Statistical Analysis

We first performed a series of homogeneity of variance tests (*F*-tests) on *Z*-scores derived from any two of the three groups (the paretic, the contralateral, and the control) for each of the three examined muscles/positions. Then, a series of Student’s *t*-tests were performed to compare the *Z*-scores derived from both the paretic muscle group and the control muscle group for each of three examined muscles/positions. Moreover, a two-way repeated-measure analysis of variance (ANOVA) was applied to the *Z*-scores with both sides (two observation levels: contralateral and paretic) and muscles/positions (three observation levels: BB, FDI, and APB) considered as within-subject factors. When necessary, *post hoc* pairwise multiple comparisons with a Bonferroni correction were used. The level of statistical significance was set to *p* < 0.05 for all analyses. All statistical analyses were completed using SPSS software (ver. 16.0, SPSS Inc., Chicago, IL, USA).

## Results

Figure 2 shows the results of the CI-area plot and the *Z*-scores for the signals of all three muscles examined on the contralateral and the paretic sides of all stroke subjects and on the dominant sides of all control subjects. In the CI-area plots, we observed that all the control data were distributed over the normal area (spanning within ±2.5 times the SE of the linear regression), while some epochs from the paretic and contralateral muscles were scattered outside the normal range. In particular, a portion of the epochs from the paretic muscles were found to be distributed beyond or below the normal range. Furthermore, epochs from the paretic muscles were always distributed at a narrower horizontal range when compared with those from the contralateral muscles and the control muscles.

Of all the control subjects, the *Z*-scores derived from the BB muscle (0.00 ± 1.00, mean ± SD), the FDI muscle (0.00 ± 1.00), and the APB muscle (−0.13 ± 0.99) scored within the predefined normal range from −2.5 to +2.5. In contrast, on the paretic side of all stroke subjects, the *Z*-scores were reported to be −1.33 ± 2.83 for paretic BB muscles, 0.23 ± 2.54 for paretic FDI muscles, and 0.068 ± 2.28 for paretic APB muscles. Some subjects displayed *Z*-scores outside the normal range. This was true for each of the three paretic muscles examined. On the contralateral side of all stroke subjects, the *Z*-scores were −1.30 ± 1.11 for the contralateral BB muscle, −1.41 ± 1.46 for the contralateral APB muscle, and 0.67 ± 1.28 for the contralateral FDI muscle.

The homogeneity of variance test revealed that paretic muscles had significant larger variances in *Z*-scores than the control muscles (*p* < 0.05) for each of the three muscles examined. In contrast, both the contralateral muscles and the control muscles exhibited homogeneity of variance for the *Z*-scores, and no significant differences were observed between them for any of the three examined muscles (*p* > 0.05). The Student’s *t*-tests revealed no significant differences in *Z*-scores between the paretic muscles and the control muscles (*p* > 0.05). In addition, the two-way repeated-measure ANOVA only yielded an overall significant effect of muscle/position (*F* = 6.04, *p* = 0.008), while the side-related effect (*F* = 0.299, *p* = 0.595) and the interaction (*F* = 2.071, *p* = 0.15) between both factors were found to be insignificant. The FDI muscle was found to yield higher *Z*-scores than any of the other two muscles, and statistical significance was obtained by *post hoc* pairwise comparisons (*p* = 0.006 for FDI versus BB muscle; *p* = 0.008 for FDI versus APB muscle). No statistical significance was found between the BB muscle and the FDI muscle (*p* = 0.329).

In particular, on the paretic side, one BB muscle from the subject S1, four FDI muscles from S1, S7, S11, and S12, and two APB muscles from S1 and S7, all had *Z*-scores greater than +2.5. These were diagnosed as being neurogenic changes. At the same time, four BB muscles from S4, S7, S10, and S12, two FDI muscles, and two APB muscles both from S5 and S8 all had *Z*-scores below −2.5. For these cases, myopathic abnormalities were reported. The paretic muscles that are not mentioned above had *Z*-scores within the normal range. Moreover, on the contralateral sides, two BB muscles from S4 and S3, in addition to two APB muscles from S5 and S3, had *Z*-scores below −2.5. Meanwhile, two FDI muscles from S2 and S5 had *Z*-scores above +2.5. The other remaining contralateral muscles were all reported to be normal.

## Discussion

In this study, we examined the FDI muscle, APB muscle, and BB muscle on both sides of all stroke subjects, in addition to the dominant sides of all control subjects using the CI method. The CI method was employed because of its reported diagnostic power in discriminating neuromuscular changes. Each paretic muscle examined showed diverse CI alternations when compared with the corresponding control muscle. Moreover, abnormalities in the two distal muscles (the FDI muscle and the APB muscle) have been obviously consistent, while this has not been the case for the proximal muscle (the biceps) when compared to distal muscles at the individual level. Regardless, abnormalities have also been reported on the contralateral side using the CI analysis.

Another reason for applying the CI method was its simple and convenient protocol as a result of manipulating and examining surface EMGs. Aside from its noninvasive aspects, the protocol does not involve accurate measurements of muscle force with a load cell, which is regarded to be tedious and time consuming (19–22). Although previous studies reported that accurate force measurements were crucial for providing substantial information (4) during routine clinical EMG examination, clinical electrophysiologists offer appropriate resistance to the tested muscle to estimate its level of activation (28–30). The use of this approach allows this study to have potentially wide applications in clinical practice.

This study confirms the previous report that CI analysis of surface EMG signals is capable of revealing complex neuromuscular changes occurring in paretic muscles following a stroke (29). For each examined muscle, some stroke subjects showed abnormally high *Z*-scores on the paretic side, indicating neuropathic changes. The abnormal increase in CI values in paretic muscles may be attributed to MU loss, reorganization of the MU architecture [including fiber-type grouping (10, 14)], and muscle fiber reinnervation (21, 23, 24, 32, 33), impairment of MU control properties [such as compression of MU recruitment threshold (18, 21, 34)], an increase in MUAP synchronization (35–37), and a reduction in MU firing rates (37–39). All of these factors are very likely to occur after a stroke as reported by previous studies. For some paretic muscles, we observed an abnormal decrease in CI leading to *Z*-scores that were lower than the predefined normal range, indicating myopathic changes. A CI decrease means that a flatter and denser surface EMG was very likely due to muscle fiber atrophy (9, 40, 41) and a selective degeneration of the large and superficial MUs (41–43). Both phenomena have been reported in stroke studies. For the other paretic muscles, their *Z*-scores fell within the normal range. However, their experience of substantial muscle weakness needs to be carefully considered. Two primary reasons may relate to the “normal” CI examination. One reason is a combination or canceling effect of both neurogenic and myopathic processes. The other is a deficit of the descending central drive. In the event of upper motor neuron lesions, the lower motor neurons and the muscles themselves might still function more or less normally without significant degeneration (25). Therefore, the paretic muscles that do not display EMG abnormalities still suffer from substantial muscle weakness.

It is worth noting that in the original CI method, CI decreases are mainly attributed to myopathic changes. The differential loss of relatively larger MUs is regarded as leading to the CI decreases in this study, but it is only a factor indicating neurogenic changes. This limits the traditional distinction between neurogenic and myopathic changes using the CI method. Therefore, it is necessary to supplement and even revise the way CI results are interpreted in order to draw diagnostic conclusions given the findings from this stroke study. Furthermore, we find that muscle fiber atrophy and the differential loss of larger MUs are both factors potentially contributing to the CI decrease. In the early phases since stroke onset (which is true for a majority of stroke subjects in this study), however, there is limited chance for the former factor to occur (9, 40). Therefore, the differential loss of larger MUs is regarded as the primary reason for the abnormal CI decrease observed in our study.

Across three different muscles on the paretic side, the two distal muscles showed almost consistent results, whereas the corresponding proximal muscle had obviously inconsistent CI scores for the same stroke subjects. This is a really interesting finding that needs to be further explained. The anatomical innervation structure would be one reason. It is well known that the FDI and APB muscles are dominated by the ulnar nerve and the median nerve, respectively, and both originate from the C8 andT1 spinal cord segments. In contrast, the BB muscle is dominated by the musculocutaneous nerve derived from the C5, C6, and C7 spinal cord segments. The difference in spinal cord segments and nerve root levels may contribute to diverse paretic muscle changes across both distal and proximal muscles at the individual subject level. This implies that the pathological changes as a result of the hemisphere brain damage have significant structural characteristics.

Different muscular structures and their original characteristics may be another reason. For example, the tissues (such as the fat or skin) between the electrode and the targeted muscle are considered to be a volume conductor likely to filter out high-frequency components of surface EMG signals. This effect of the volume conductor is always enhanced by the proximal BB muscles because of its thicker tissue composition compared to the other two distal muscles. Therefore, the ability for the CI-based surface EMG to discern some neurogenic changes (such as muscle fiber reinnervation and MU architecture reorganization) might be compromised because they likely result in reinnervated, enlarged MUs that specifically contribute to high-frequency EMG activities. As mentioned above, these neurogenic processes should lead to a CI increase. When various processes occur involving both concurrent CI increases and decreases, the reduced impact of the processes leading to a CI increase may collectively decrease CI values. This could account for the overall CI decrease in BB muscle when compared with the other two distal muscles.

Additionally, both of the distal muscles are regarded as dominantly carrying small MUs composed of slow-twitch, low-force muscle fibers, which are suitable for fine motor control. In contrast, the BB muscle consists of a greater portion of larger MUs with faster muscle fibers that generate larger forces. It has been discussed above that the selective loss of larger and superficial MUs is potentially taking place in paretic muscles, leading to a decrease in the CI. Therefore, the BB muscle is hypothesized to lose its larger MUs because of the impact of a stroke, whereas this factor may not be evident for smaller and more distal muscles. This hypothesis is partially supported by our experimental data. Part B of Figure 2 show that 4 out of the 12 paretic BB muscles that had abnormally decreased CI and *Z*-scores, many more than that number (2) of distal paretic FDI and APB muscles reported CI abnormalities with *Z*-scores below −2.5. Thus, a greater proportion of larger MUs in the proximal BB muscle likely degenerate following a stroke compared to small distal muscles like the FDI and APB.

It is surprising that the CI-revealed neuromuscular abnormalities appeared in some muscles on the contralateral side of stroke subjects regardless of the muscle position. This finding is inconsistent with our current understanding that the contralateral muscles are considered to be neurologically intact. The muscle abnormalities on the contralateral side might be attributed to impaired interactions in the partially damaged brain (44, 45). Furthermore, motor control in muscle that is compensatory on the contralateral side after hemiparesis is another reason (46). For example, changes in control strategies in the motor neuron pool may lead to altered MU recruitment strategies and firing properties. This finding suggests the necessity of delivering a treatment to the contralateral muscles during stroke rehabilitation.

The main limitation of this study was the limited choice of either proximal or distal muscles. Future investigations will be continuously performed with more muscles in order to draw stronger conclusions. Moreover, although CI measurements can provide a valuable assessment of paretic muscles, more delicate analyses are required to further discriminate the underlying complex factors contributing to the observed CI patterns (47). Modeling (18, 48) might be an effective approach to detect the CI marker’s sensitivity with respect to individual factors, which will help to obtain a more accurate interpretation of abnormal CI findings. All of these efforts will help us better understand muscle pathologies that arise after a stroke, which is the prerequisite for designing and developing effective stroke rehabilitation protocols.

## Ethics Statement

This study was approved by the Medical Ethic Review Committee at the First Affiliated Hospital of Anhui Medical University (FAHAMU, Hefei, Anhui Province, China). The written consent was obtained from all subjects before experiments.

## Author Contributions

WT analyzed the data, interpreted the results, and wrote the first draft of the manuscript. XZ designed the study, including data collection, analysis, interpretation, and a substantial revision of the manuscript. XT and SC participated in data collection and interpretation. XG and XC participated in data analysis, interpretation, and manuscript revision. All authors approved the final version of the manuscript.

## Conflict of Interest Statement

The authors declare that this research was conducted in the absence of any commercial or financial relationships that could be construed as a potential conflict of interest.
